# Epidemiological and Clinical Features of SARS-CoV-2 Variants Circulating between April–December 2021 in Italy

**DOI:** 10.3390/v14112508

**Published:** 2022-11-12

**Authors:** Alessia Lai, Annalisa Bergna, Carla Della Ventura, Stefano Menzo, Bianca Bruzzone, Fabio Sagradi, Francesca Ceccherini-Silberstein, Alessandro Weisz, Nicola Clementi, Gaetano Brindicci, Ilaria Vicenti, Lolita Sasset, Sara Caucci, Benedetta Corvaro, Silvia Ippoliti, Carla Acciarri, Vanessa De Pace, Leonardo Lanfranchi, Maria C. Bellocchi, Giorgio Giurato, Roberto Ferrarese, Antonella Lagioia, Daniela Francisci, Martina L. Colombo, Samuel Lazzarin, Matilde Ogliastro, Maria R. Cappelletti, Marco Iannetta, Francesca Rizzo, Carlo Torti, Maurizio Fumi, Morena d’Avenia, Stefano Brusa, Francesca Greco, Angela Menchise, Vittoria Letizia, Emilia Vaccaro, Carmen R. Santoro, Chiara Fraccalvieri, Sophie Testa, Luca Carioti, Teresa Rocco, Annalisa Saracino, Annamaria Cattelan, Massimo Clementi, Loredana Sarmati, Agostino Riva, Massimo Galli, Spinello Antinori, Gianguglielmo Zehender, SARS-CoV- ITALIAN RESEARCH ENTERPRISE–(SCIRE) Collaborative Group

**Affiliations:** 1 Department of Biomedical and Clinical Sciences, Università degli Studi di Milano, 20174 Milan, Italy; 2 Virology Unit, Department of Biomedical Sciences and Public Health, Polytechnic University of Marche, 60131 Ancona, Italy; 3 Hygiene Unit, IRCCS AOU San Martino-IST, 16132 Genoa, Italy; 4 Unit of Infectious Diseases, Azienda Socio Sanitaria Territoriale Cremona, 26100 Cremona, Italy; 5 Department of Experimental Medicine, University of Rome Tor Vergata, 00133 Rome, Italy; 6 Laboratory of Molecular Medicine and Genomics, Department of Medicine, Surgery and Dentistry ‘Scuola Medica Salernitana’, University of Salerno, 84084 Salerno, Italy; 7 Laboratory of Microbiology and Virology, Università “Vita-Salute” San Raffaele, 20158 Milan, Italy; 8 Infectious Diseases Unit, University of Bari, 70121 Bari, Italy; 9 Department of Medical Biotechnologies, University of Siena, 53100 Siena, Italy; 10 Infectious Diseases Unit, Azienda Ospedale Università di Padova, 35128 Padova, Italy; 11 Department of Medicine and Surgery, Clinic of Infectious Diseases, Santa Maria della Misericordia Hospital, University of Perugia, 06123 Perugia, Italy; 12 Department of Health Sciences (DISSAL), University of Genoa, 16126 Genoa, Italy; 13 Infectious Disease Unit, Department of Systems Medicine, University of Rome Tor Vergata, 00133 Rome, Italy; 14 Infectious and Tropical Disease Unit, Department of Medical and Surgical Sciences, Magna Graecia University of Catanzaro, 88100 Catanzaro, Italy; 15 UOC Patologia Clinica, AO San Pio Benevento, 82100 Benevento, Italy; 16 UOSVD di Citopatologia e Screening, Department of Laboratory Medicines, 70131 Bari, Italy; 17 Department of Translational Medical Sciences, Università Federico II, 80138 Naples, Italy; 18 UOC Microbiologia e Virologia, PO Cosenza, 87100 Cosenza, Italy; 19 Microbiology and Virology Laboratory, A.O.R. San Carlo Potenza, 85100 Potenza, Italy; 20 UOSD Genetics and Molecular Biology, AORN Sant’Anna e San Sebastiano di Caserta, 81100 Caserta, Italy; 21 Molecular Biology Units, AOU ‘S. Giovanni di Dio e Ruggi d’Aragona’ Università di Salerno, 84131 Salerno, Italy

**Keywords:** variants circulation, SARS-CoV-2, Italy, epidemiology

## Abstract

SARS-CoV-2 is constantly evolving, leading to new variants. We analysed data from 4400 SARS-CoV-2-positive samples in order to pursue epidemiological variant surveillance and to evaluate their impact on public health in Italy in the period of April–December 2021. The main circulating strain (76.2%) was the Delta variant, followed by the Alpha (13.3%), the Omicron (5.3%), and the Gamma variants (2.9%). The B.1.1 lineages, Eta, Beta, Iota, Mu, and Kappa variants, represented around 1% of cases. There were 48.2% of subjects who had not been vaccinated, and they had a lower median age compared to the vaccinated subjects (47 vs. 61 years). An increasing number of infections in the vaccinated subjects were observed over time, with the highest proportion in November (85.2%). The variants correlated with clinical status; the largest proportion of symptomatic patients (59.6%) was observed with the Delta variant, while subjects harbouring the Gamma variant showed the highest proportion of asymptomatic infection (21.6%), albeit also deaths (5.4%). The Omicron variant was only found in the vaccinated subjects, of which 47% had been hospitalised. The diffusivity and pathogenicity associated with the different SARS-CoV-2 variants are likely to have relevant public health implications, both at the national and international levels. Our study provides data on the rapid changes in the epidemiological landscape of the SARS-CoV-2 variants in Italy.

## 1. Introduction

The ongoing global coronavirus Disease 2019 (COVID-19) pandemic has caused significant mortality and morbidity [1], requiring unprecedented efforts to develop novel vaccines and strategies for fighting COVID-19. Despite its limited intrinsic genetic variability, the huge number of infections has led to the evolution of SARS-CoV-2 in an expanding array of new variants. While the driver of this evolution is certainly immune escape, the new emerging variants may differ from those previously circulating also in terms of increased transmissibility and virulence. These variations can affect diagnostic testing reliability, available treatment effectiveness, and vaccine efficacy [2,3]. Currently, the spike (S) protein is the region most affected by mutations, and the circulating SARS-CoV-2 variants are classified on the basis of variations in the S protein compared to the ancestral strain [4]. Among the growing number of SARS-CoV-2 variants documented worldwide during the pandemic, previously identified variants of concern (VOCs), such as Alpha (B.1.1.7/20I), Beta (B.1.351/20H), Gamma (B.1.1.28/P1), and Delta (B.1.617.2), have been associated with increased transmissibility, mortality, and decreased susceptibility to neutralising antibodies induced by vaccination or previous infection [2,3,5,6,7,8].

Among these, the Beta variant has been significantly more resistant to neutralisation from the sera of convalescent or vaccinated subjects, mainly because of the presence of the E484K mutation [9]. The P.1 and Delta variants are related to increased transmissibility and virulence and reduced sensitivity to neutralising antibodies [10,11,12,13]. The Delta variant has displayed additional mutations in the spike protein, such as P681R, E484Q, and L452R, with the last two being associated with immune escape [14]. Different sublineages previously classified as variants of interest (VOI), i.e.**,** Kappa (B.1.617.1/21B), Iota (B.1.526/21F), Lambda (C.37/21G), and Mu (B.1.621/21H), and also previously prevalent VOCs, have been declassified as they become “extinct” and cease to impact the overall epidemiological situation (https://www.who.int/activities/tracking-SARS-CoV-2-variants (accessed on 3 November 2022)).

Currently, Omicron is the only VOC circulating worldwide (https://gisaid.org/, accessed on 3 November 2022), largely causing breakthrough infections [15].

In light of its widespread transmission worldwide and its observed viral diversity, the WHO added a new category to the variant tracking system, termed “Omicron sub-variants under monitoring” (VUM), in order to alert public health authorities globally on variants requiring prioritised attention and monitoring (BA.4.6, BA.5, BJ.1, XBB, BA.2.3.20, and BA.2.75) (https://www.who.int/activities/tracking-SARS-CoV-2-variants, accessed on 3 November 2022).

Our previous analysis [16] reported on the data obtained in several Italian regions involved in SARS-CoV-2 variant monitoring in the period of October 2020–March 2021. The aim of the present study is to continue epidemiological surveillance in Italy and to analyse the clinical data of infected subjects in the period ranging from April to December 2021.

## 2. Materials and Methods

### 2.1. Sample Collection and Study Design

This retrospective observational study included 4400 SARS-CoV-2-positive nasopharyngeal swabs obtained from COVID-19-positive patients referred to Italian Centres participating in the SCIRE (SARS-CoV-2 Italian Research Enterprise) collaborative group (see the map for the involved centres’ distribution all over the Italian territory: https://arcg.is/19f9mn2, accessed on 3 November 2022) during the period from 1 April to 31 December 2021. The distribution of samples through the different months was as follows: 357 (April), 267 (May), 184 (June), 304 (July), 443 (August), 409 (September), 323 (October), 764 (November), and 1349 (December).

All the genotypic, demographic, epidemiologic, and clinical data used for the analyses were collected at each centre as a part of routine variant surveillance or for research purposes.

This study was conducted in accordance with the principles of the 1964 Declaration of Helsinki and approved by the Sacco Hospital ethics committee (protocol n. 47866, 9 September 2020).

### 2.2. Virus Amplification and Sequencing

Genotypic data were obtained by using different methods: RT-PCR variant screening assays (*n* = 2535), spike Sanger (*n* = 938), next-generation sequencing (NGS, *n* = 46), and whole-genome sequencing (WGS, *n* = 881). These data, stratified according to the different methodologies and timing of sample collection, are shown in Table 1.

SARS-CoV-2 swabs were collected from the respiratory tract of individuals who were either hospitalised or tested in screening programs. Viral RNA was extracted using different commercial kits, such as the QIAsymphony DSP Virus/Pathogen Midi kit on the QIAsymphony automated platform (QIAGEN, Hilden, Germany), the NucleoMag 96 Virus (Macherey-Nagel, Dueren, Germany) on automated KingFisherTM ml Magnetic Particle Processors (Thermo Fisher Scientific, Waltham, MA, USA), and manually with the QIAamp Viral RNA Mini Kit (QIAGEN, Hilden, Germany). RT-PCR genotyping assays were performed using the TaqPath COVID-19 test (Thermo Fisher Scientific, MA, USA), the COVID-19 Ultra Variant Catcher (Clonit Srl., Milan, Italy), the Allplex SARS-CoV-2 Variants (Arrow Diagnostics Srl., Genoa, Italy), the multiplexed RT-qPCR developed by English consortium (https://www.protocols.io/view/multiplexed-rt-qpcr-to-screen-for-sars-cov-2-b-1-1-br9vm966?version_warning=no, accessed on 3 November 2022), or homemade protocols. The spike sequences were obtained using homemade protocols. Full genome sequences were obtained with different protocols, by a modified version of the Artic Protocol (https://artic.network/ncov-2019, accessed on 3 November 2022) using the Illumina DNA Prep and the IDT ILMN DNA/RNA Index kit (Illumina, San Diego, CA, USA) or by the CleanPlex**^®^** SARS-CoV-2 Panel (Paragon Genomics Inc., Hayward, CA, USA). Sequencing was performed on the Illumina iSeq (*n* = 99), Miseq (*n* = 644), and Nextseq (*n* = 138) platforms for all samples. The results were mapped and aligned to the reference genome obtained from GISAID (https://www.gisaid.org/, accession ID: EPI_ISL_406800, accessed on 3 November 2022), using the Geneious Prime software v. 11.1 (http://www.geneious.com, Biomatters, Auckland, New Zealand, accessed on 3 November 2022) or BWA-mem, and rescued using Samtools alignment/Map (Hinxton, UK) (v. 1.9).

The SARS-CoV-2 lineage was attributed to all sequences using the Pangolin COVID-19 Lineage Assigner v. 4.1.1 (https://pangolin.cog-uk.io/, accessed on 3 November 2022) and Nextclade v. 2.4.1 (https://clades.nextstrain.org/, accessed on 3 November 2022). Mutations were identified using Nextclade.

### 2.3. Statistical Analysis

Descriptive analyses of the demographic and clinical data are presented as a median and an inter-quartile range (IQR) when continuous and as a frequency and a proportion (%) when categorical. To compare normally distributed, non-normally distributed continuous, and categorical variables, parametric tests (t-test and ANOVA), nonparametric tests (Mann–Whitney and Kruskal–Wallis), and the Pearson χ_2_ test (or Fisher exact test, when necessary) were used, respectively. Significance was established at *p* < 0.05. Data analysis was performed using the IBM SPSS Statistics version 25.

## 3. Results

### 3.1. Characteristics of the Study Population

The samples were collected from several Italian centres located in Apulia (*n* = 85), Liguria (*n* = 375), Campania (*n* = 56), Calabria (*n* = 25), Lombardy (*n* = 1145), Basilicata (*n* = 13), Umbria (*n* = 144), Marche (*n* = 2311), Lazio (*n* = 192), Veneto (*n* = 53), and also the Republic of San Marino (*n* = 1). There were slightly more females than men (51.4%, 1582/3078), and the median age was 47 years old (y.o.) (IQR: 29–63). Significant differences were observed in the median age over different months (*p* < 0.001), with the lowest median age reported in July (35 y.o., IQR: 22–55) and the highest in April (57 y.o., IQR: 35–72).

Among the subjects with available data, 51.9% (*n* = 507/979) had received at least one dose of a COVID-19 vaccine, and 2.3% (*n* = 12/532) reported previous exposure to SARS-CoV-2.

At the time of testing, mild infections were the most frequent (2330/2965, 78.6%), followed by moderate/severe infections requiring hospitalisation (474/2965, 16%). Only 4.7% (138/2965) of patients were asymptomatic. Deaths were reported in 23 patients among the subjects with known outcomes (0.8%).

Statistically significant differences in clinical status were observed in patients <60 y.o. and ≥60 y.o. Globally, the resulting proportions of hospitalised subjects (44.2% vs. 55.7%; *p* < 0.001) and deaths (14.4% vs. 85.7%; *p* < 0.001) were higher in older compared to younger patients. Of note, asymptomatic status was more prevalent in subjects under the age of 60; on the contrary, deaths were observed only in patients over 60 y.o., with the exception of two unvaccinated patients in September and one patient vaccinated with only a single dose in November.

### 3.2. Lineages and Clades

Overall, the main circulating variant, representing more than 76.2% of the total sequences (3333/4375), was the Delta B.1.617.2 lineage (60.5%, *n* = 2648) together with its descendants (15.7%, *n* = 685, including AY.4, AY.4.1, AY.4.2, AY.4.2.1, AY.4.2.3, AY.4.3, AY.4.6, AY.4.7, AY.4.9, AY.5, AY.5.4, AY.9, AY.9.1, AY.9.2, AY.10, AY.20, AY.23, AY.25, AY.26, AY.33, AY.34, AY.34.1, AY.36, AY.39, AY.42, AY.43, AY.44, AY.46.4, AY.46.6, AY.46.6.1, AY.48, AY.51, AY.53, AY.54, AY.58, AY.61, AY.68, AY.71, AY.73, AY.75, AY.82, AY.92, AY.91.1, AY.98, AY.98.1, AY.102, AY.103, AY.106, AY.112, AY.112.2, AY.118, AY.116, AY.119.2, AY.120, AY.122, AY.122.2, AY.122.3, AY.124, AY.124.1, AY.125, AY.126, AY.127, and AY.129), followed by the Alpha variant (B.1.1.7 lineage, 13.3%, *n* = 583) and the Omicron variant (B.1.1.529 lineage, 5.3%, *n* = 230), with its sublineages, BA.1 (*n* = 219), BA.1.1 (*n* = 3), and BA.1.17.2 (*n* = 8).

A proportion of 2.9% of the samples (*n* = 127) was represented by the Gamma variant, including P.1 (*n* = 79), P.1.1 (*n* = 36), P.1.15 (*n* = 2), and P.1.6 (*n* = 10). About 1% of the samples were of the lineage B.1.1 and its descendants (*n* = 47), the Eta variant (*n* = 10, B.1.525), the Beta variant (*n* = 12, B.1.351), the Iota variant (*n* = 8, B.1.526), the Mu variant (*n* = 10, B.1.621), and the Kappa variant (*n* = 1, B.1.617.1). Five cases of XF recombinants were also observed.

Considering clade classification (*n* = 2109), the most prevalent were 21J (*n* = 756, 35.8%) and 20I (*n* = 583, 27.6%), followed by 21A (*n* = 267, 12.7%) and 21K (*n* = 235, 11.1%). Clade 21I showed a prevalence of 2.4% (*n* = 51) while clades 20A (*n* = 8), 20B (*n* = 16), 20D (*n* = 16), 20E.EU1 (*n* = 9), 20H (*n* = 12), 21B (*n* = 1), 21D (*n* = 10), 21F (*n* = 8), and 21H (*n* = 10) had a prevalence lower than 1%.

### 3.3. Lineages and Clades over Time

Over the study period, the prevalence of the Alpha variant (B.1.1.7/20I) significantly (*p* < 0.001) decreased from 77.8% (*n* = 273) in April 2021 to 0.7% (*n* = 3) in August 2021 until its disappearance. Only one single case was reported in November 2021 in Lombardy.

The Gamma variant (P.1 and its descendant/20J) remained stable in the first three months with a prevalence of 12.5% (*n* = 44), 17.1% (*n* = 45), and 19% (*n* = 35), in April, May, and June, respectively. The last cases were observed in July (*n* = 3); one of these (lineage P.1.15) was identified in a 25-year-old hospitalised patient travelling from Argentina to Apulia.

The previously circulating lineage, B.1.1, and its descendants were present at low prevalence only until August 2021, decreasing from 6.8% in April (*n* = 24) to 0.7% (*n* = 2) in July. One case of B.1.177 (20E.EU1) was observed in August in Lombardy.

The last case of the Beta variant (B.1.351/20H) was identified in August, never reaching a prevalence of above 2%.

Only a few cases of the Eta variant (B.1.525/21D) were reported until June in Marche when it reached the highest prevalence (2.7%, *n* = 5). Two cases of the Iota variant (B.1.526/21F) were detected in April in Marche and Liguria, with an additional six cases in May and June in Liguria.

The Mu variant (B.1.621/21H) was first detected in Marche (*n* = 1) in April; since then, a limited number of cases were reported until July, with the highest percentage in June (3.2%; *n* = 6) in Marche. A single case of the Kappa variant (B.1.617.1/21B) was observed in May in Liguria in a young (25 years old) asymptomatic subject.

Starting from the middle of June, the Delta variant (B.1.617.2) was observed in the following Regions: Liguria, Lazio, Veneto, and Marche (9.7%; *n* = 18), together with 19 (10.3%) sub-lineages (AY.42, AY.61, AY.106, and AY.122) identified in Liguria, Apulia, Campania, and Marche. Among these, a hospitalised subject reported a recent trip to Afghanistan. The prevalence of the Delta variant and its sublineages rapidly increased, reaching the totality of cases in September and in October, while in June and July, clades 21A and 21J showed similar proportions (8.2% vs. 10.9% in June and 44% vs. 32.7% in July) and in the following months, clade 21J became prevalent. The prevalence of clade 21J significantly increased over time (*p* < 0.001), remaining around 70% from August (74.1%; 140/189) to November (72.5%; 140/193), while decreasing in December (45.6%; 239/524). On the other hand, clade 21I always maintained a low prevalence, with the highest frequency (9.5%; *n* = 16) reported in July.

The first cases of the Omicron variant (B.1.1.529/21K) were detected in Lombardy (*n* = 4), with the first sequence reported in the city of Cremona (Lombardy) on 5 November, 2021. Its prevalence reached 16.8% of cases in December 2021.

Of note, its prevalence during the month of December increased steadily: to 0.4% (29 November–5 December), to 0.5% (6–12 December), to 5% (13–19 December), to 16.8% (20–26 December), and to 61.7% (27–31 December). In this month, in addition to the BA.1 lineage (*n* = 215), we observed the presence of sub-lineages BA.1.1 (*n* = 3) and BA.1.17.2 (*n* = 8).

**Figure 1:**
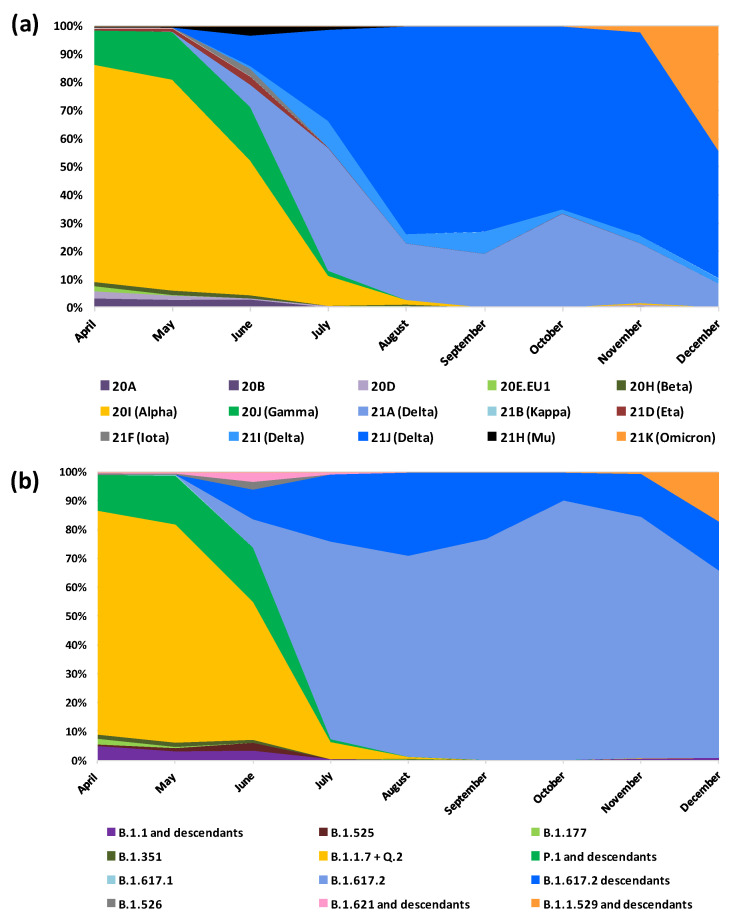
Dynamics of the SARS-CoV-2 epidemic in Italy. (**a**) Variant prevalence in terms of clades; (**b**) variant prevalence in terms of lineages.

Five cases of XF recombinants were identified in December in the Marche Region, predominantly in vaccinated and hospitalised subjects (80%). Figure 1 shows the main viral variants and clades over time.

The median age significantly differed according to the different viral variants (*p* < 0.001), with younger subjects more affected by the Omicron variant (40 years, IQR: 24–58) compared to the other lineages and sub-lineages (Alpha: 51 years, IQR: 32–67; Gamma: 51 years, IQR: 40–64; Delta: 46 years, IQR: 29–61; Delta descendants: 49 years, IQR: 29–68).

**Figure 2:**
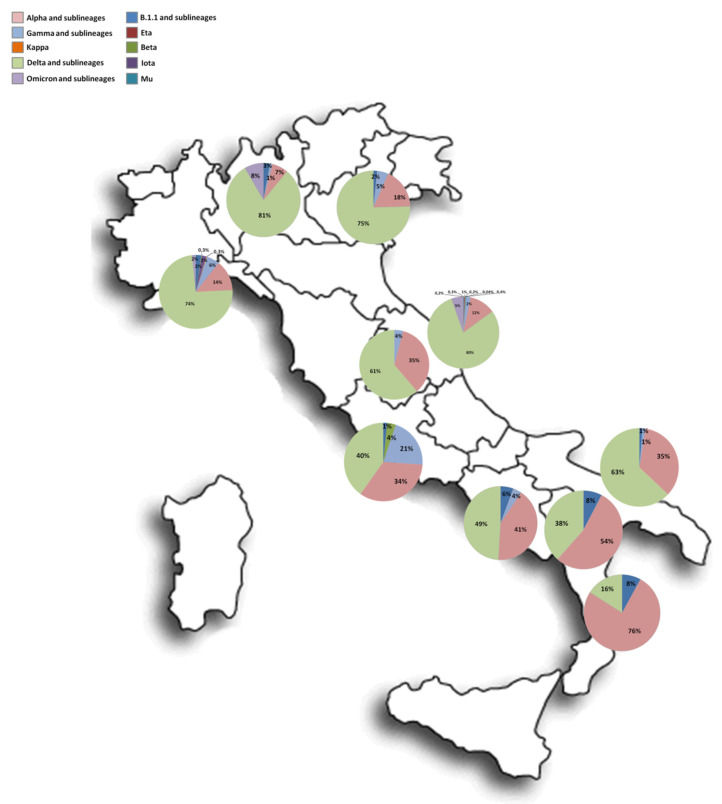
Spatial distribution of lineages in different regions.

Figure 2 illustrates the distribution of lineages in different Italian regions.

### 3.4. Vaccinated vs. Unvaccinated

**Figure 3:**
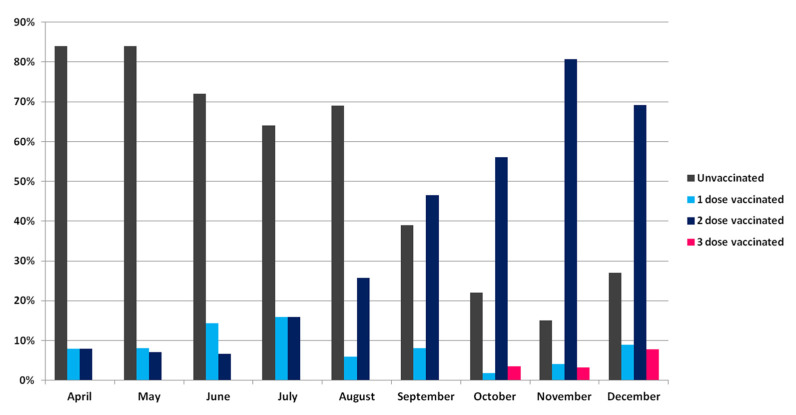
Proportion of unvaccinated and vaccinated subjects over time.

We observed a significant increase in the proportion of vaccinated subjects receiving at least one dose of the COVID-19 vaccine, rising from 15.8% to 73.2% (*p* < 0.001) over the study period, with the highest percentage reached in November 2021 (85.2%, 115/135) (Figure 3).

Overall, 48.2% (472/979) of infected subjects had not been vaccinated, and the unvaccinated were younger compared to the vaccinated (47 vs. 61 years). Most vaccinated subjects reported two doses of a vaccine (78.4%, 366/467), of which 50.7% with the BNT162b2 vaccine and 17.4% (*n* = 80) had been vaccinated with a single dose (60% with BNT162b2 vaccine). Only 21 subjects (4.5%) had received three doses (42.8%, of which always with the BNT162b2 vaccine), and their proportion increased from October (*n* = 2, 0.4%) to December (*n* = 15, 3.2%). The median time from vaccination to SARS-CoV-2 infection was 28 (IQR: 12–120), 150 (IQR: 120–180), and 13.5 days (IQR: 6–41.2) for subjects who had received one, two, or three doses of a vaccine, respectively.

The median age of patients who had received three doses of a vaccine was significantly (*p* = 0.018) higher compared to that of those who had received one or two doses (70, IQR: 57.5–82 vs. 60, IQR: 45–77 and 59 years, IQR: 39–69).

Twelve patients experienced a documented reinfection; 11 were diagnosed among the unvaccinated subjects, while the remaining one occurred in a subject who had received a single dose of a vaccine. They had a median age of 54 years (IQR: 41-63), and three of them were hospitalised. Reinfections were distributed as follows: 3, 3, 1, 2, 1, 1, and 1 in April, May, June, July, August, September, and December, respectively. Until June, all patients were re-infected with the Alpha variant, while from August, the Delta variant (21J) was reported among reinfections.

**Figure 4:**
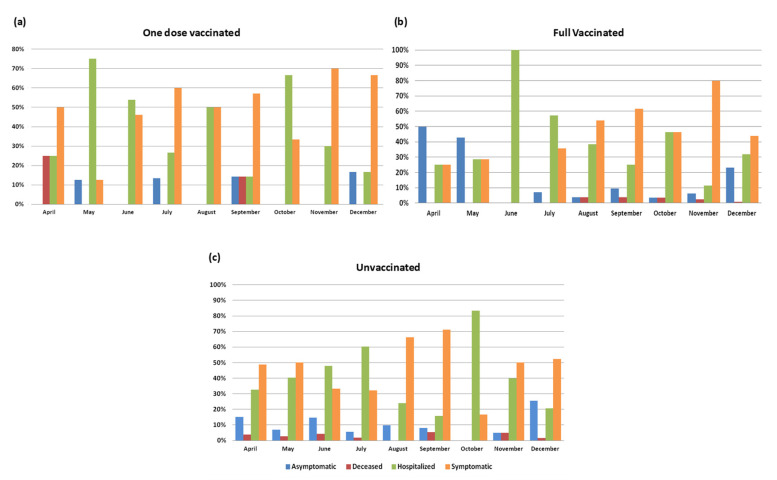
Clinical status/outcome in (**a**) subjects vaccinated with one dose, (**b**) subjects vaccinated with two or three doses, and (**c**) unvaccinated subjects during the study period.

Among subjects with a known vaccination status (*n* = 881), no differences were observed between the vaccinated and the unvaccinated concerning clinical status (12.7% vs. 11.8%, 53.3% vs. 49.7%, 31.6% vs. 35.9%, and 0.2% vs. 0.3%, in regard to asymptomatic, symptomatic, hospitalised, and dead subjects, respectively) (Supplementary Table S1). Moreover, no differences were observed considering single-dose vaccinated or fully vaccinated and unvaccinated subjects (Figure 4).

When stratifying patients according to age, a statistically significant higher proportion of hospitalisations and deaths were observed in the vaccinated subjects over 60 y.o. compared to the vaccinated subjects <60 y.o. (76.5% and 88.9%, *p* < 0.001); importantly, deaths were significantly higher in the unvaccinated versus the vaccinated individuals (81.8%, *p* < 0.001) (Supplementary Table S2). Of note, at the end of the data collection (April 2022), 91% (*n* = 429) of the unvaccinated subjects had not received any doses yet.

### 3.5. Clinical Status and Vaccination According to Variants

The viral variants also significantly correlated to clinical status in the subset of patients with known clinical status and viral variants (*n* = 2899, *p* = 0.001). Symptomatic patients were observed more frequently (59.5%) amongst those infected by the Delta variant compared to the other variants. Subjects harbouring the Gamma variant (*n* = 80) showed the highest frequency of asymptomatic status (21.6%), but oddly, also of deaths (5.4%).

**Figure 5:**
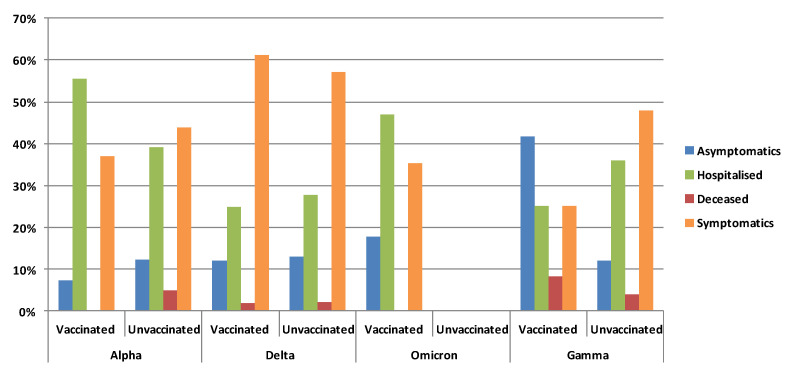
Proportion of clinical status in the vaccinated and unvaccinated subjects infected with the different variants.

In the subset of patients with known vaccination and clinical status (*n* = 772), significant differences according to viral variant were observed only among the vaccinated subjects (*n* = 393, *p* < 0.001). The vaccinated symptomatic patients were more frequent (61.1%) among those with the Delta variant infection, while the hospitalised subjects were more frequent in those infected with the Alpha variant (55.6%). As already observed in the whole cohort, the vaccinated subjects with the Gamma variant presented the highest proportion of asymptomatic status (41.7%) but also of deaths (8.3%). No death was observed in the vaccinated subjects carrying the Alpha and the Omicron variants (Figure 5).

The Omicron variant was only observed in vaccinated subjects, of which 47% had been hospitalised.

Concerning vaccination status, the majority of infected subjects (63%) with the Alpha variant had been vaccinated with one dose, while 58.3%, 76.5%, and 83.5% of those with the Gamma, Omicron, and Delta variants had received two doses. Only 11.8% and 3.7% of subjects carrying the Omicron and Delta variants, respectively, reported a booster dose. A similar proportion of deaths was associated with the Alpha and Gamma variants (4.9% and 4%, respectively).

## 4. Discussion

In this study, based on the characterisation of the SARS-CoV-2 strains circulating in Italy, complemented by demographics and partially by clinical data, we provide a clear picture of the spread of the SARS-CoV-2 variants in the Italian territories, highlighting how the replacement of the previously dominant variants of concern has dictated the subsequent epidemic waves in the country.

The timing of this study mostly overlaps with the emergence and the spread of the Delta variant and its descendants throughout Italy at the beginning of summer 2021 and offers a unique and well-characterised cohort of hospitalised and non-hospitalised patients covering all the Italian territories, with the exclusion of the islands.

Among the variants rapidly causing concern, B.1.1.7 (Alpha clade) and B.1.351 (Beta clade)/P.1 (Gamma clade) emerged in our country in December 2020 and January 2021 [16], respectively, while the first cases of B.1.617.2 (Delta clade) were described in June 2021, reaching a prevalence of 20.1% in this same month, as was also reported in the European and national surveys [17,18,19,20,21].

All of these variants were clearly characterised by increased transmissibility compared with the preceding lineages, starting with the original lineage, which was detected at very low levels (<1% of total sequences) in the study period. As a consequence, B.1.617.2 + AY was the most prevalent variant in our population, followed by B.1.1.7 (76.2% and 13.3%). Most of the epidemiological success of these new variants can be attributed to their ability to overcome previous natural or vaccine-induced herd immunity, in addition to an intrinsic increase in their transmissibility.

The SARS-CoV-2’s most recent VOC, Omicron, has rapidly replaced the SARS-CoV-2 Delta variant in most European countries, including Italy, starting from December 2021 [22]. Omicron, with its potential to evade the host’s immune response induced by previous infections or vaccination, is considered more adaptable and transmissible than its predecessors. Immunity acquired after previous infection or vaccination is less effective against Omicron than against other variants, but the risk of severe COVID-19 is low [23].

Our study was interrupted at the end of December when only the first sublineage of Omicron (BA.1) was present; however, such a lineage was observed only in vaccinated subjects, mostly with asymptomatic or mild disease (53%).

The first detection of the Omicron variant in our country was dated at the beginning of November 2021 in Lombardy, followed by its rapid and wide diffusion in December (from 0.4% to 61.7% in 4 weeks).

On 20 December 2021, the institutional “flash survey” conducted in Italy showed that the Delta variant was still predominant, and the prevalence of the Omicron variant was 21.0% (18), in line with our data, indicating a prevalence of 16.8%.

Our data confirm that older age is associated with an increased risk of severe COVID-19 clinical course and death, also in vaccinated individuals, as reported in several studies [24,25].

Surprisingly, our results did not reveal differences between vaccinated and unvaccinated subjects concerning their clinical status of the disease. This is certainly due to the fact that the population was not randomly selected among infected subjects, but most subjects were included as patients independently referred to hospitals (and therefore, affected by more severe symptoms), therefore missing most mild/asymptomatic infections. In fact, the study population showed a low proportion of mild/asymptomatic infections (12% in our data). By contrast, a significant association was found between SARS-CoV-2 lineages and COVID-19 presentation, even if, for some variants, a low number of cases was considered.

Significant associations were found only in vaccinated subjects; specifically, hospitalisation and death showed a higher incidence in those over 60 y.o. and in subjects with the Alpha and Gamma variants, respectively.

This could possibly be explained by the fact that when these variants circulated, most of the vaccinated patients had only received a single dose. The observed median time from vaccination to infection was similar to that reported in recent work [26] and possibly due to the early production of anti-SARS-CoV-2 facilitating antibodies.

This study presents some limitations. Our study does not analyse the course and the outcome of the infections and does not consider the Ct (cycle threshold of real-time PCR) levels at diagnosis. Albeit a large number of samples was included in the study, these are not proportionally distributed among regions in relation to their total population and the related number of confirmed cases. Moreover, data were missing for many subjects, particularly concerning vaccination and data related to the first three months of 2021, characterised by the Alpha variant circulation, were not evaluated in the present analysis because they have already been discussed in previous work [16].

The COVID-19 pandemic continues to represent a global health crisis. Analyses of the epidemiology and clinical outcomes associated with the SARS-CoV-2 variants have important public health implications, both nationally and internationally. Our report complements previous analyses by providing further data on the rapid change in the epidemiological landscape of SARS-CoV-2 variants in Italy, revealing that in addition to the known variants of concern, other minor variants circulate and contribute to the epidemic.

Our study provides strong evidence consolidating the notion that since the beginning of the epidemic, local lineage replacements are associated with new local epidemic waves [16,27]. This is in line with recent work indicating that the re-increase in SARS-CoV-2 incidence could be due to the emergence of new variants rather than a rebound of previous viral genotypes [28].

Our data also reveal that during each main variant epidemic wave, the expansion of minor variants (descendant lineages) contributes significantly to the epidemics, creating “sub-waves” that likely extend the epidemic course of their parent lineage. Furthermore, independent of where these subvariants were generated, they may spread to other geographical areas with the potential to reverberate globally. In this context, the role of continuous SARS-CoV-2 genomic monitoring to follow local viral evolution in real time appears of the utmost importance.

In addition, the association between the genomic and clinical data allowed us to evaluate the role of viral variants in vaccinated people with respect to the severity of the disease.

## Figures and Tables

**Table 1:** Description of analysed data.

	April (*n* = 357)	May (*n* = 267)	June (*n* = 184)	July (*n* = 304)	August (*n* = 443)	September (*n* = 409)	October (*n* = 323)	November (*n* = 764)	December (*n* = 1349)	Total (*n* = 4400)
RT-PCR	162	57	25	118	248	219	211	514	981	2535
NGS WG	134	149	81	59	109	77	32	76	164	881
NGS Spike	42	1	0	0	0	0	0	0	3	46
Sanger Spike	19	60	78	127	86	113	80	174	201	938

RT-PCR: real-time PCR; NGS: next-generation sequencing; WGS: whole-genome sequencing.

## Data Availability

Whole-genome sequences were submitted to GISAID.

## Supplementary Materials

The following supporting information can be downloaded at: https://www.mdpi.com/article/10.3390/v14112508/s1, Supplementary information. Table S1: Number of individuals with known clinical status/outcome stratified according to vaccination data. Table S2: Number of subjects stratified according to clinical and vaccination status and age over time.
