# Supplementary material for: Epidemiological and Clinical Features of SARS-CoV-2 Variants Circulating between April–December 2021 in Italy
Source: Viruses. 2022 Nov 12;14(11):2508. doi: 10.3390/v14112508 (PMC9699621; doi:10.3390/v14112508)
Supplement: Supplementary file 1 [file viruses-14-02508-s001.zip › Supplementary Information.pdf]

Supplementary Information

SCIRE collaborative group: Claudia Balotta[1], Chiara Resnati[1], Mario Corbellino[1], Alessandro Occhionero[2], Katia Marinelli[2], Valentina Ricucci[3], Alexander Domnich[3], Andrea Orsi[3], Federica Stefanelli[3], Nadia Randazzo[3], Giada Garzillo[3], Lorenzo Piermatteo[5], Leonardo Duca[5], Stefano D'Anna[5], Greta Marchegiani[5], Nicasio Mancini[7], Sofia Sisti[7], Maurizio Zazzi[9], Lia Fiaschi[9], Lucrezia Calandrino[10], Massimo Andreoni[13], Laura Campogiani[13], Mirko Compagno[13], Luigi Coppola[13], Angela Maria Antonia Crea[13], Giuseppe De Simone[13], Andrea Di Lorenzo[13], Ludovica Ferrari[13], Vincenzo Malagnino[13], Tiziana Mulas[13], Benedetta Rossi[13], Ilaria Spalliera[13], Simona Tedde[13], Elisabetta Teti[13], Pietro Vitale[13], Marta Zordan[13], Giovanni Matera[14], Enrico Maria Trecarichi[14], Alessandro Russo[14], Angela Quirino[14], Nadia Marascio[14], Salvatore Rotundo[14].
