# Supplementary material for: Epidemiological and Clinical Features of SARS-CoV-2 Variants Circulating between April–December 2021 in Italy
Source: Viruses. 2022 Nov 12;14(11):2508. doi: 10.3390/v14112508 (PMC9699621; doi:10.3390/v14112508)
Supplement: Supplementary file 1 [file viruses-14-02508-s001.zip › Supplementary Table S1.pdf]

Supplementary Table S1. Number of individuals with known clinical status/outcome stratified according vaccination data.

Clinical status	Vaccination status	Month								
		April	May	June	July	August	September	October	November	December
Asymptomatics	Partially Vaccinated	4	4	0	3	1	6	1	5	30
	Full Vaccinated	4	3	0	1	1	5	1	5	29
	Unvaccinated	12	5	7	3	7	3	0	1	16
Deceased	Partially Vaccinated	2	0	0	0	1	3	1	2	1
	Full Vaccinated	0	0	0	0	1	2	1	2	1
	Unvaccinated	3	2	2	1	0	2	0	1	1
Hospitalized	Partially Vaccinated	4	8	13	12	13	14	17	12	41
	Full Vaccinated	2	2	6	8	10	13	13	9	40
	Unvaccinated	26	29	23	32	17	6	10	8	13
Symptomatics	Partially Vaccinated	6	3	6	14	17	36	15	70	59
	Full Vaccinated	2	2	0	5	14	32	13	63	55
	Unvaccinated	39	36	16	17	47	27	2	10	33
