# Supplementary material for: Epidemiological and Clinical Features of SARS-CoV-2 Variants Circulating between April–December 2021 in Italy
Source: Viruses. 2022 Nov 12;14(11):2508. doi: 10.3390/v14112508 (PMC9699621; doi:10.3390/v14112508)
Supplement: Supplementary file 1 [file viruses-14-02508-s001.zip › Supplementary Table S2.pdf]

**Supplementary Table S2**. Number of subjects stratified according to clinical and vaccination status and age overtime.

| | | | Month | | | | | | | | | |
|---|---|---|---|---|---|---|---|---|---|---|---|---|
| | | **Age** | **April** | **May** | **June** | **July** | **August** | **September** | **October** | **November** | **December** | **Total** |
| **Asymptomatics** | Vaccinated | <60y | 1 | 2 | / | 1 | 1 | 4 | 1 | 3 | 18 | **31** |
| | | ≥60y | 3 | 3 | 1 | 2 | / | 2 | / | 2 | 12 | **25** |
| | Unvaccinated | <60y | 9 | 5 | 4 | 3 | 5 | 3 | / | 1 | 15 | **45** |
| | | ≥60y | 3 | / | / | / | 2 | / | / | / | 1 | **6** |
| **Deceased** | Vaccinated | <60y | / | / | / | / | / | 1 | / | / | / | **1** |
| | | ≥60y | 2 | / | / | / | / | 2 | 1 | 2 | 1 | **8** |
| | Unvaccinated | <60y | / | / | / | / | / | 1 | / | 1 | / | **2** |
| | | ≥60y | 2 | 2 | 2 | 1 | / | 1 | / | / | 1 | **9** |
| **Hospitalized** | Vaccinated | <60y | 1 | / | 2 | 4 | 5 | 2 | 1 | 4 | 4 | **23** |
| | | ≥60y | 3 | 8 | 3 | 8 | 8 | 6 | 16 | 7 | 16 | **75** |
| | Unvaccinated | <60y | 6 | 16 | 15 | 22 | 10 | 3 | 4 | 5 | 5 | **86** |
| | | ≥60y | 18 | 13 | 5 | 10 | 7 | 3 | 5 | 3 | 7 | **71** |
| **Symptomatics** | Vaccinated | <60y | 3 | / | 6 | 13 | 16 | 18 | 11 | 36 | 35 | **138** |
| | | ≥60y | 3 | 2 | / | 1 | 1 | 16 | 4 | 34 | 24 | **85** |
| | Unvaccinated | <60y | 25 | 26 | 3 | 15 | 44 | 27 | 1 | 5 | 29 | **175** |
| | | ≥60y | 14 | 10 | 2 | 2 | 3 | / | 1 | 5 | 4 | **41** |
| | | **Total** | **93** | **87** | **43** | **82** | **102** | **89** | **45** | **108** | **172** | |
